# Supplementary material for: CGR-CUSUM: a continuous time generalized rapid response cumulative sum chart
Source: Biostatistics. 2022 Sep 19;25(1):253–69. doi: 10.1093/biostatistics/kxac041 (PMC10939399; doi:10.1093/biostatistics/kxac041)
Supplement: kxac041_Supplementary_Data [file kxac041_supplementary_data.zip › kxac041-suppl_data/biosts-21444-File003.pdf]

Biostatistics (2022), **0**, 0, *pp.* 1–14
doi:10.1093/biostatistics/output

Supplementary materials to CGR-CUSUM: A Continuous time Generalized Rapid response Cumulative Sum chart

DANIEL GOMON*

Mathematical Institute, Leiden University, Leiden, The Netherlands d.gomon@math.leidenuniv.nl

HEIN PUTTER

Department of Biomedical Data Sciences, Leiden University Medical Centre, Leiden,The Netherlands

ROB G.H.H. NELISSEN

Department of Orthopaedic Surgery, Leiden University Medical Centre, Leiden,The Netherlands

STÉPHANIE VAN DER PAS

Department of Epidemiology and Data Science, Amsterdam UMC, Vrije Universiteit Amsterdam, Amsterdam, The Netherlands

1. Some proofs

To prove our main result stated in Theorem 4.1, we first need to derive some properties of other quantities, which will be done in this section.

Lemma 1 Assume that f_i^θ and h_i^θ are non-negative and Borel measurable. Define $\Lambda(t) = \sum_{i \geqslant 1} \Lambda_i(t)$. Then:

$$\begin{aligned}\mathbb{E}[d\Lambda(u)] &= e^{-\theta}\psi\mathbb{E}_{Z_i}\left[F_i^\theta(u)\right]du \\ &=: \gamma_u du\end{aligned}$$

and:

$$\mathbb{E}[\Lambda(t)] = \int_0^t \gamma_u du$$

*To whom correspondence should be addressed.

© The Author 2022. Published by Oxford University Press. All rights reserved. For permissions, please e-mail: journals.permissions@oup.com

with ψ the rate of arrivals and $e^{\theta}\Lambda_i(t)$ the true risk-adjusted subject specific cumulative intensity of failure at the institute of interest.

Proof. We consider a hospital with hazard rate e^{θ} times the baseline hazard rate. We define:

$$U_{CGI}(t) := \hat{\theta}(t)N(t) + (e^{\hat{\theta}(t)} - 1)\Lambda(t) \tag{1.1}$$

where:

$$\hat{\theta}(t) = \max\left\{0, \ln\left(\frac{N(t)}{\Lambda(t)}\right)\right\}.$$

Assume that patients arrive according to a homogeneous Poisson process with rate $\psi > 0$. We choose to consider the lifetime of all patients up until the time of failure (or censoring). The first step is to calculate the expected value of $d\Lambda(u)$:

$$\begin{aligned}
\mathbb{E}[d\Lambda(u)] &= \mathbb{E}\left[\sum_{i\geqslant 1} Y_i(u)h_i(u - S_i)du\right] \\
&= e^{-\theta}\mathbb{E}\left[\sum_{i\geqslant 1} \mathbb{1}\{S_i \leqslant u, X_i \geqslant u - S_i\}e^{\theta}h_i(u - S_i)du\right].
\end{aligned}$$

Here we use that $T_i = S_i + X_i$. Then using the law of total expectation twice (conditioning first on Z_i and then on S_i):

$$\begin{aligned}
\mathbb{E}[d\Lambda(u)] &= e^{-\theta}\sum_{i\geqslant 1}\mathbb{E}_{S_i}\left[\mathbb{E}_{Z_i}\left[\mathbb{E}\left[\mathbb{1}\{S_i \leqslant u\}\mathbb{1}\{X_i \geqslant u - S_i|Z_i\}e^{\theta}h_i(u - S_i|Z_i)du\right]|S_i\right]\right] \\
&= e^{-\theta}\sum_{i\geqslant 1}\mathbb{E}_{S_i}\left[\mathbb{1}\{S_i \leqslant u\}\mathbb{E}_{Z_i}\left[\mathbb{E}\left[\mathbb{1}\{X_i \geqslant u - S_i|Z_i\}\right]e^{\theta}h_i(u - S_i|Z_i)du|S_i\right]\right] \\
&= e^{-\theta}\sum_{i\geqslant 1}\mathbb{E}_{S_i}\left[\mathbb{1}\{S_i \leqslant u\}\mathbb{E}_{Z_i}\left[\mathbb{P}\left(X_i \geqslant u - S_i|Z_i\right)h_i^{\theta}(u - S_i|Z_i)du|S_i\right]\right] \\
&= e^{-\theta}\sum_{i\geqslant 1}\mathbb{E}_{S_i}\left[\mathbb{1}\{S_i \leqslant u\}\mathbb{E}_{Z_i}\left[S_i^{\theta}\left(u - S_i|Z_i\right)h_i^{\theta}(u - S_i|Z_i)du|S_i\right]\right] \\
&= e^{-\theta}\sum_{i\geqslant 1}\mathbb{E}_{S_i}\left[\mathbb{1}\{S_i \leqslant u\}\mathbb{E}_{Z_i}\left[f_i^{\theta}(u - S_i|Z_i)|S_i\right]\right]du \\
&= e^{-\theta}\sum_{i\geqslant 1}\int_0^u \mathbb{E}_{Z_i}\left[f_i^{\theta}(u - x)\right]\psi\frac{e^{-\psi x}(\psi x)^{i-1}}{(i-1)!}dxdu \\
&= e^{-\theta}\psi\int_0^u \mathbb{E}_{Z_i}\left[f_i^{\theta}(u - x)\right]e^{-\psi x}\sum_{i\geqslant 1}\frac{(\psi x)^{i-1}}{(i-1)!}dxdu.
\end{aligned}$$

We obtain using Fubini's Lemma that:

$$\mathbb{E}[d\Lambda(u)] = e^{-\theta}\psi\int_0^u \mathbb{E}_{Z_i}\left[f_i^{\theta}(u - x)\right]du \tag{1.2}$$

$$= e^{-\theta}\psi\mathbb{E}_{Z_i}\left[F_i^{\theta}(u)\right]du. \tag{1.3}$$

We define, similarly to above, the notation:

$$\gamma_u := e^{-\theta}\psi\mathbb{E}_{Z_i}[F_i^\theta(u)] > 0. \tag{1.4}$$

We assumed that f_i^θ and h_i^θ are non-negative and Borel measurable, therefore using Fubini's Lemma once again we obtain that $\mathbb{E}[\Lambda(t)] = \int_0^t \gamma_u du$. □

2. Fisher Information

We derive the Fisher information contained in all observations:

Lemma 2 The Fisher information in all observations at time $t > 0$ is given by:

$$I(\theta, t) = \psi \int_0^t \mathbb{E}_{Z_i}\left[F_i^\theta(k)\right] dk. \tag{2.5}$$

Proof. The likelihood function for all observations is given by:

$$L(\theta|t) = \prod_{i \geqslant 1} \left(e^\theta d\Lambda_i(t)\right)^{dN_i(t)} e^{-e^\theta \Lambda_i(t)}.$$

The log likelihood ratio is then given by:

$$l(\theta|t) = \sum_{i \geqslant 1}(dN_i(t))(\theta + \ln(d\Lambda_i(t))) - e^\theta \sum_{i \geqslant 1} \Lambda_i(t).$$

Taking the derivative w.r.t. θ yields:

$$\frac{\partial l(\theta|t)}{\partial \theta} = \sum_{i \geqslant 1} dN_i(t) - e^\theta \sum_{i \geqslant 1} \Lambda_i(t).$$

And the second derivative:

$$\frac{\partial^2 l(\theta|t)}{\partial \theta^2} = -e^\theta \sum_{i \geqslant 1} \Lambda_i(t).$$

Finally, the Fisher information is given by:

$$I(\theta, t) = -\mathbb{E}\left[-e^\theta \sum_{i \geqslant 1} \Lambda_i(t) \mid \theta\right] \tag{2.6}$$

$$= e^\theta \mathbb{E}_\theta\left[\Lambda(t)\right] \tag{2.7}$$

$$= e^\theta e^{-\theta}\psi \int_0^t \mathbb{E}_{Z_i}\left[F_i^\theta(k)\right] dk \tag{2.8}$$

$$= \psi \int_0^t \mathbb{E}_{Z_i}\left[F_i^\theta(k)\right] dk \tag{2.9}$$

where we have used our result from Lemma 1. □

3. Convergence of MLE

In this section we reiterate a result from Hoadley (1971) and transform it so it becomes usable for the problem at hand.

Lemma 3 Let $t > 0$ and suppose we have n patients. Let $\{f_i^\theta, i = 1, ..., n\}$ meet conditions (N1)-(N9) of Hoadley (1971). Then, as $n \to \infty$:

$$\sqrt{n}(\hat{\theta}(t) - \theta) \xrightarrow{d} \mathcal{N}\left(0, \frac{1}{\overline{I(\theta, t)}}\right)$$

with $\overline{I(\theta,t)} = \frac{I(\theta,t)}{n}$ and $I(\theta, t)$ the Fisher information in all observations at time t. Moreover, assuming that $n = \psi \cdot t$ we have that:

$$\sqrt{t}\left(\hat{\theta}(t) - \theta\right) \xrightarrow{d} \mathcal{N}\left(0, \frac{1}{\psi \cdot \overline{I(\theta, t)}}\right)$$

as $t \to \infty$.

Proof. Section 4 of Hoadley (1971) tells us that under conditions $(N1) - (N9)$ as stated in the article our first statement holds. Most of these conditions are likely to hold when h_i^θ is continuous and twice differentiable. Finally, as patients arrive according to a Poisson process with rate ψ, it is reasonable (especially when ψ is large) to assume that $n = t \cdot \psi$, as we expect to see ψ patients arrive per time unit. Using this relation we then have that $n \to \infty$ implies that $t \to \infty$ (remember that ψ is constant), therefore using the properties of a normal distribution we obtain the second statement. □

4. Proof of Theorem 2.1

In this section we prove the main theorem in our article. This proof uses the results stated in the previous sections.

Theorem 4.1 Suppose that patients arrive according to a Poisson process with rate ψ and let some other regularity conditions hold. It follows that when $\theta > 0$:

$$\sqrt{t}\left(CGI(t) - (\theta + \exp(-\theta) - 1)I(\theta, t)\right) \xrightarrow{d} \mathcal{N}\left(0, t\theta^2 I(\theta, t)\right)$$

and when $\theta = 0$:

$$t \cdot CGI(t) \xrightarrow{d} \Gamma\left(0.5, t\right)$$

where

$$I(\theta, t) = \psi \int_0^t \mathbb{E}_{Z_i}\left[F_i^\theta(k)\right] dk$$

is the Fisher information in all observations at time t.

Proof. **1. When $\boldsymbol{\theta > 0}$:** Assuming that $N(t) = e^{\hat{\theta}(t)}\Lambda(t)$ we can write:

$$\begin{aligned} CGI(t) &= \hat{\theta}(t)N(t) - (e^{\hat{\theta}(t)} - 1)\Lambda(t) \\ &= \hat{\theta}(t)e^{\hat{\theta}(t)}\Lambda(t) - (e^{\hat{\theta}(t)} - 1)\Lambda(t). \end{aligned}$$

Now assume that $\Lambda(t)$ is a constant with value $\mathbb{E}[\Lambda(t)] = \int_0^t \gamma_u du = e^{-\theta} I(\theta, t)$ (using Lemma 1). We can write:

$$CGI(t) = \hat{\theta}(t)e^{\hat{\theta}(t)}e^{-\theta}I(\theta, t) - (e^{\hat{\theta}(t)} - 1)e^{-\theta}I(\theta, t).$$

Then consider the function ϕ defined as:

$$\phi(x) = xe^x e^{-\theta} I(\theta, t) - e^x e^{-\theta} I(\theta, t) + e^{-\theta} I(\theta, t)$$

and note that $\phi(\hat{\theta}(t)) = CGI(t)$. Additionally, we find that ϕ is differentiable at θ with derivative:

$$\phi'(x) = e^{-\theta} I(\theta, t) x e^x.$$

Then by the delta method (see section 7 of van der Vaart (2007)) and the result of Lemma 3 we obtain that:

$$\sqrt{n}\left(\phi(\hat{\theta}(t)) - \phi(\theta)\right) \xrightarrow{d} \mathcal{N}\left(0, \frac{(\phi'(\theta))^2}{I(\theta, t)}\right)$$

as $n \to \infty$ which reduces to:

$$\sqrt{t}\left(CGI(t) - \left(\theta + e^{-\theta} - 1\right) I(\theta, t)\right) \xrightarrow{d} \mathcal{N}\left(0, t\theta^2 I(\theta, t)\right)$$

when $t \to \infty$ using a similar argument ($n = \psi \cdot t$) as in the proof of Lemma 3.

2. When $\boldsymbol{\theta = 0}$: In this case we can no longer use the delta method to determine the distribution of $CGI(t)$ as $\phi'(\theta) = \phi'(0) = 0$. Luckily we can use the second-order delta method (see Theorem 5.5.26 of Casella and Berger (2002)). Note that:

$$\phi''(x) = (x + 1)e^x e^{-\theta} I(\theta, t)$$

and $\phi''(0) = I(\theta, t)$. Now the second-order delta-method in combination with Lemma 3 tells us that:

$$n\left(CGI(t) - \phi(0)\right) \xrightarrow{d} \frac{1}{I(\theta,t)} \frac{\phi''(0)}{2}\chi_1^2$$

as $n \to \infty$ which simplifies to:

$$t \cdot CGI(t) \xrightarrow{d} \frac{t}{2}\chi_1^2 = \Gamma\left(\eta = \frac{1}{2}, \nu = t\right)$$

as $t \to \infty$, using the shape/scale(η/ν) parametrization of the Gamma distribution (and using that $n = \psi \cdot t$). □

4.1 *A martingale approach*

A result similar to Theorem 4.1 can be proved using a Martingale approach.

Theorem 4.2 As t becomes large, the expected value of the CGI-CUSUM chart is approximated by:

$$\mathbb{E}[CGI(t)] \approx (\theta e^{\theta} - e^{\theta} + 1)\Lambda(t)$$

Proof.

The CGI-CUSUM can be written as:

$$CGI(t) = \hat{\theta}(t)N(t) - \left(e^{\hat{\theta}(t)} - 1\right)\Lambda(t) \tag{4.10}$$

$$= \left(\hat{\theta}(t) - \theta\right)N(t) - \left(e^{\hat{\theta}(t)} - e^{\theta}\right)\Lambda(t) + \theta N(t) - e^{\theta}\Lambda(t) + \Lambda(t) \tag{4.11}$$

Lemma 3 tells us that $\sqrt{t}\left(\hat{\theta}(t) - \theta\right)$ converges to a normal distribution with mean zero and strictly decreasing variance $\frac{1}{\psi I(\theta,t)}$ as t becomes large. Applying the delta method we obtain that $\sqrt{t}\left(e^{\hat{\theta}(t)} - e^{\theta}\right)$ converges to a normal distribution with mean zero and strictly decreasing variance $\frac{e^{2\theta}}{\psi I(\theta,t)}$ as t becomes large. As t becomes large, the first two terms of Equation (4.11) will therefore become small. We can then approximate:

$$CGI(t) \approx \theta N(t) - e^{\theta}\Lambda(t) + \Lambda(t)$$

When $\theta > 0$, $e^{\theta}\Lambda(t)$ is the compensator of $N(t)$ and $M(t) := N(t) - e^{\theta}\Lambda(t)$ is a zero-mean martingale as $M(0) = 0$ per construction (see Aalen *and others* (2008) Section 2.2.5). We rewrite CGI(t):

$$CGI(t) \approx \theta N(t) - \theta e^{\theta}\Lambda(t) + \theta e^{\theta}\Lambda(t) - e^{\theta}\Lambda(t) + \Lambda(t)$$

$$= \theta M(t) + (\theta e^{\theta} - e^{\theta} + 1)\Lambda(t)$$

Again, we assume $\Lambda(t)$ is constant with value $\mathbb{E}[\Lambda(t)] = e^{-\theta} I(\theta, t)$ (using Lemma 1) to obtain:

$$
\begin{aligned}
CGI(t) &\approx \theta M(t) + (\theta e^{\theta} - e^{\theta} + 1) e^{-\theta} I(\theta, t) \\
&= \theta M(t) + (\theta + e^{-\theta} - 1) I(\theta, t)
\end{aligned}
$$

Then conditioning on the history at time zero $\mathcal{F}_0$ we obtain:

$$
\begin{aligned}
\mathbb{E}\left[CGI(t) | \mathcal{F}_0\right] &= \theta \cdot 0 + (\theta + e^{-\theta} - 1) I(\theta, t) \\
&= (\theta + e^{-\theta} - 1) I(\theta, t)
\end{aligned}
$$

where we have used the martingale property $\mathbb{E}[M(t)|\mathcal{F}_s] = M(s)$ for all $s \leqslant t$. □

Equating this expression to $h > 0$ and solving for t we obtain the same expression for the average run length of the CGR-CUSUM as using the method above.

5. Approximate ARL of BK-CUSUM

Corollary 1 Suppose θ_1 is chosen such that $\exp(\theta_1)/\exp(\theta) < \theta_1 + \exp(-\theta)$. We find an approximate average run length $\widehat{\text{ARL}}_{BK}(\theta, h)$ by solving the following equation for t:

$$
\left(\theta_1 + \exp(-\theta) - \frac{\exp(\theta_1)}{\exp(\theta)}\right) I(\theta, t) = h. \tag{5.12}
$$

Proof. Assuming that $N(t) = e^{\theta}\Lambda(t)$ we can write:

$$
\begin{aligned}
BK(t) &= \theta_1 N(t) - (e^{\theta_1} - 1)\Lambda(t) \\
&= \theta_1 e^{\theta}\Lambda(t) - (e^{\theta_1} - 1)\Lambda(t).
\end{aligned}
$$

From Lemma 1 we know that $\mathbb{E}[\Lambda(t)] = \int_0^t \gamma_u du = e^{-\theta} I(\theta, t)$. Taking the expected value of $BK(t)$ we then obtain:

$$
\begin{aligned}
\mathbb{E}\left[BK(t)\right] &= \theta_1 e^{\theta} e^{-\theta} I(\theta, t) - (e^{\theta_1} - 1) e^{-\theta} I(\theta, t) \\
&= (\theta_1 + e^{-\theta} - \frac{e^{\theta_1}}{e^{\theta}}) I(\theta, t)
\end{aligned}
$$

By equating the expected value of this expression to h we can find an approximate average run length for the BK-CUSUM. As $I(\theta, t)$ is non-negative, this is only possible for control limits $h > 0$ when $(\theta_1 + e^{-\theta} - \frac{e_1^{\theta}}{e^{\theta}}) > 0$. □

6. Standard simulation procedure

- Step 1: Generating a training (in control) data set with N hospitals.
  1. Choose (parametric) null cumulative baseline hazard rate or determine from existing data (for example, using R package survival (Therneau, 2020)).
  2. Generate patient arrival times in the required time frame using Poisson arrivals with rate ψ.
  3. (Optional) Resample patient characteristics from data set.
  4. Determine (risk-adjusted) survival times for every patient using above chosen cumulative baseline hazard rate using the method described by (Bender *and others*, 2005).
  5. Repeat 2-4 N times. Combine into single data set.
- Step 2: Determining a suitable control limit h.
  1. Determine a (parametric) cumulative baseline hazard rate using the generated in control data set. Optionally, use the cumulative hazard rate from step 1.
  2. Construct the charts on the training data set.
  3. Determine control limit h such that required restrictions (on sensitivity or ARL under the null) are met for the collection of the constructed charts.
- Step 3: Generate test (out of control) data set.
  1. Follow step 1 with $\theta > 0$ as required.
- Step 4: Evaluate charts on test data set
  1. Construct the charts on the test data with the control limit determined in step 2.
  2. Approximate required quantities (such as ARL, sensitivity, specificity) from these charts.

7. Calculation of the Fisher information

There are two hurdles in the calculation of the Fisher information found in Section 2. First of all we need to calculate $\mathbb{E}_{\mathbf{Z}_i}\left[F_i^{\theta}(s)\right]$. We propose a method to tackle this calculation in Section 7.1 as well as some examples for the PVF family of distributions. The second hurdle is then calculating the

resulting integral $\int_0^t \mathbb{E}_{\mathbf{Z}_i}\left[F_i^\theta(s)\right] ds$. For this step, an assumption must be made for the hazard rate. As an example, we calculate a closed form expression for the Fisher information for exponential failure times in Section 7.2.

7.1 *Risk-adjustment*

An approach which can be used to calculate the expected value of the risk-adjusted cumulative distribution function is by using Laplace transforms.

Lemma 4 Assuming that $e^{\mathbf{Z}_i^\top \boldsymbol{\beta}} \sim U$ with U a general distribution, the Fisher information at time t is given by:

$$I(\theta, t) = \psi t - \psi \int_0^t \mathcal{L}(H^\theta(s)) ds$$

with $\mathcal{L}(H^\theta(s)) = \mathbb{E}\left[e^{-H^\theta(s)U}\right]$ the Laplace transform of U and $H^\theta(k) = e^\theta H(k)$ the cumulative hazard rate multiplied by the hazard ratio e^θ.

Proof. Note that we can write:

$$\begin{aligned} I(\theta, t) &= \psi \int_0^t \mathbb{E}_{\mathbf{Z}_i}\left[F_i^\theta(s)\right] ds \\ &= \psi \int_0^t \mathbb{E}_{\mathbf{Z}_i}\left[1 - S_i^\theta(s)\right] ds \\ &= \psi t - \psi \int_0^t \mathbb{E}_{\mathbf{Z}_i}\left[e^{-H^\theta(s) e^{\mathbf{Z}_i^\top \boldsymbol{\beta}}}\right] ds \end{aligned}$$

with $H^\theta(k) = e^\theta H(k)$ the cumulative hazard rate multiplied by the hazard ratio e^θ. We assumed that $e^{\mathbf{Z}_i^\top \boldsymbol{\beta}} \sim U$ where U follows some general distribution. Then define the Laplace transform of U as follows:

$$\mathcal{L}(c) = \mathbb{E}\left[e^{-cU}\right]$$

We can then write:

$$I(\theta, t) = \psi t - \psi \int_0^t \mathcal{L}(H^\theta(s)) ds$$

□

Example: **Gamma distribution**

A common assumption is to take $U \sim \Gamma(\eta, \nu)$ (shape/scale parametrization). We then obtain:

$$I(\theta, t) = \psi t - \psi \int_0^t \left(\frac{\nu}{\nu + H^\theta(s)} \right)^\eta ds$$

Taking the mean of the covariate distribution to be equal to 1 (i.e. $\eta = \nu$) and defining $\delta = \frac{1}{\nu}$ we then obtain:

$$I(\theta, t) = \psi t - \psi \int_0^t \left(1 + \delta H^\theta(s)\right)^{-\frac{1}{\delta}} ds$$

Example: **Family of PVF distributions**

Consider the family of PVF distributions, which are distributions having a Laplace transform in the form:

$$\mathcal{L}(c; \rho, \nu, m) = \exp\left[-\rho\left\{1 - \left(\frac{\nu}{\nu + c}\right)^m\right\}\right]$$

with $\nu > 0$, $m > -1$ and $m\rho > 0$. For more information on this family of distributions, we direct the reader to Aalen *and others* (2008) Section 6.2.3. We then obtain the following expression for the Fisher information:

$$I(\theta, t) = \psi t - \psi \int_0^t \exp\left[-\rho\left\{1 - \left(\frac{\nu}{\nu + H^\theta(s)}\right)^m\right\}\right] ds$$

7.2 *Exponential failure times*

In this section we consider the case when the failure times are exponentially distributed.

Example: **Exponential distribution (no covariates)**

Suppose failure times are exponentially distributed with parameter $\lambda > 0$. We calculate the Fisher

information $I(\theta, t)$ without risk-adjustment:

$$\begin{aligned} I(\theta,t) &= \psi \int_0^t F^\theta(s) ds \\ &= \psi \int_0^t 1 - e^{-H^\theta(s)} ds = \psi \int_0^t 1 - e^{-H(s)e^\theta} ds \\ &= \psi \int_0^t 1 - e^{-\lambda s e^\theta} ds \\ &= \psi t - \psi \left[\frac{e^{-\lambda e^\theta s}}{\lambda e^\theta} \right]_0^t \\ &= \psi \left(t - \frac{1 - e^{-\lambda e^\theta t}}{\lambda e^\theta} \right) \end{aligned}$$

with $H(s)$ the cumulative hazard rate.

Example: **Exponential distribution with Gamma distributed risk-adjustment**

Suppose failure times are exponentially distributed with parameter $\lambda > 0$. Using Lemma 4 and assuming that the covariates are Gamma distributed with mean 1 and variance δ we find:

$$\begin{aligned} I(\theta,t) &= \psi t - \psi \int_0^t \left(1 + \delta \lambda s e^\theta\right)^{-\frac{1}{\delta}} ds \\ &= \psi t - \psi \left[\frac{\left(1 + \delta \lambda e^\theta s\right)^{1-\frac{1}{\delta}}}{\lambda e^\theta \left(\delta - 1\right)} \right]_0^t \\ &= \psi t - \psi \left(\frac{\left(1 + \delta \lambda e^\theta t\right)^{1-\frac{1}{\delta}} - 1}{\lambda e^\theta (\delta - 1)} \right) \end{aligned}$$

7.3 *Numerical computation*

For covariate distributions which do not have an (easy) Laplace transform or hazard rates with no closed form expression, we suggest using the relationship:

$$F_i^\theta(t) = 1 - S_i^\theta(t) = 1 - e^{-H(t)\exp(\theta)\exp\left(\mathbf{Z}_i^\top \boldsymbol{\beta}\right)}$$

with $H(t)$ the cumulative hazard rate. The resulting function can then be numerically integrated first with respect to $\exp\left(\mathbf{Z}_i^\top \boldsymbol{\beta}\right)$ and then with respect to t to obtain $I(\theta, t)$. Without risk-adjustment, $\exp\left(\mathbf{Z}_i^\top \boldsymbol{\beta}\right)$ can be left out of the equation.

8. Additional Figures and Tables

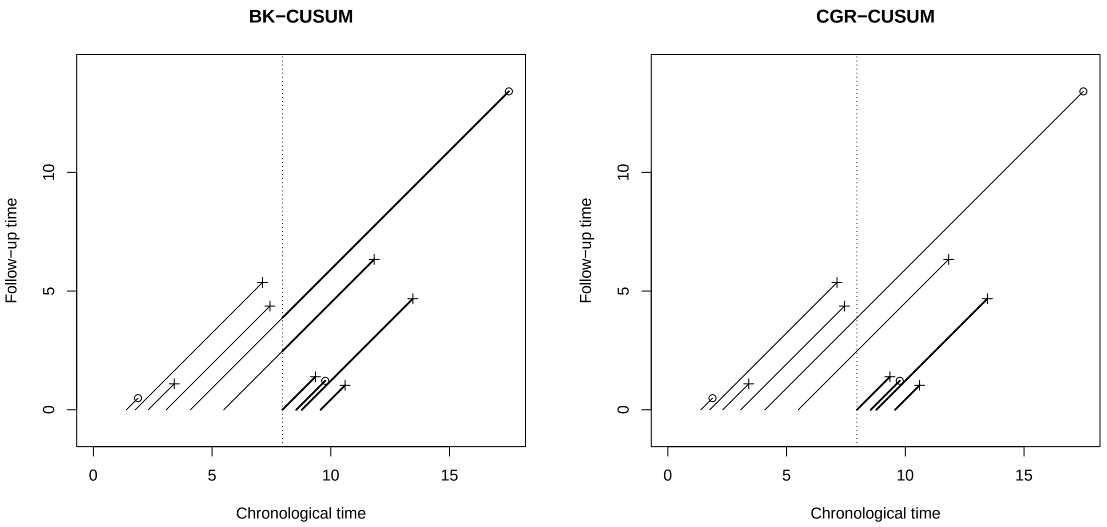

(a) Lexis diagram of BK-CUSUM (b) Lexis diagram of CGR-CUSUM

Fig. 1: Lexis diagrams of BK-CUSUM and CGR-CUSUM charts. The line segments in bold represent the information used by the chart if both charts were to detect the same change point (dotted vertical line).

References

Aalen, O. O., Borgan, Ø. and Gjessing, S. (2008). *Survival and event history analysis: A process point of view*, 1st edition. New York, NY: Springer.

Bender, R., Augustin, T. and Blettner, M. (2005). Generating survival times to simulate cox proportional hazards models. *Statistics in Medicine* **24**, 1713–1723.

Casella, G. and Berger, R. L. (2002). *Statistical inference*, 2nd edition. Pacific Grove, CA: Thomson Learning.

Hoadley, B. (1971). Asymptotic properties of maximum likelihood estimators for the independent not identically distributed case. *The Annals of Mathematical Statistics* **6**, 1977–1991.

Therneau, T. M. (2020). *A Package for Survival Analysis in R.* R package version 3.2-7 https://CRAN.R-project.org/package=survival.

van der Vaart, A. W. (2007). *Asymptotic statistics*, Cambridge Series in Statistical and Probabilistic Mathematics. Cambridge: Cambridge University Press.

Hospital nr.	Funnel plot $p = 0.95$ yearly	Bernoulli CUSUM $h = 3.5, e^{\theta} = 2$ monthly	BK-CUSUM $h = 5.1, e^{\theta} = 2$ monthly	CGR-CUSUM $h = 6.8, e^{\hat{\theta}(t)} \leqslant 6$ monthly
5	36	31		
9	36	30	21	19
13	36	15	20	21
17	36	24	15	12
19	36	31		
22	36	16	8	7
23	36	29	25	25
32	36	23	24	27
37	36	27	21	18
46	36	30	25	21
48	36	25	18	19
74	36	32	24	19
80	36	27	19	18
11	48		24	
39	48	40	40	42
42	48			
58	48		40	
87	48			
63	60	58	55	
81	60	52	59	
2	72	56	48	48
8	72	63	39	59
73	72			
4		64	54	
6		52	40	46
18		60	54	53
26		44		
29		48	39	20
35		70	43	64
41		43		33
44			71	
50		62		
55		61	59	51
60		66		
68			59	
83				39

Table 1: Detection speed of charts in months on the LROI data set. Red cells indicate that this method did not yield a detection on the corresponding hospital before 01/01/2020. Rows are sorted first according to funnel plot detection time and hospital number afterwards.

N = 97 hospitals	Median (IQR)	Range
**Continuous Variables**		
Mean age (years)	69.5 (66.8 - 70.2)	51.8 - 81.5
Mean BMI (kg/m^2)	27.2 (26.9 - 27.5)	21.2 - 28.4
**Discrete Variables**		
Gender (%)		
Female	65.3 (63.2 - 67.1)	17.5 - 100
Male	34.7 (32.9 - 36.8)	0 - 82.5
Smoking (%)		
Yes	11.6 (10 - 13.4)	0 - 18.4
No	88.4 (86.6 - 90)	81.6 - 100
ASA Classification (%)		
I	15.5 (13.1 - 20.4)	0 - 53
II	63.7 (59.4 - 68.2)	43.8 - 93.8
III-IV	19.2 (12.6 - 24.6)	0 - 50
Charnley Score (%)		
A	47.1 (41.3 - 51.1)	0 - 76.7
B1	29 (25 - 33)	7.1 - 50
B2	21.5 (19.4 - 23.4)	5.5 - 50
C	2.3 (1.2 - 3.8)	0 - 16
Diagnosis (%)		
Osteoarthritis	86.9 (83.7 - 90.3)	0 - 98.8
Not Osteoarthritis	13.1 (9.7 - 16.3)	1.2 - 100
**Statistics**		
Procedures (number)		
in 3 years	756 (454 - 1227)	0 - 2523
in 6 years	1638 (1036 - 2462)	2 - 5093
Revision (%)		
1 year	1.7 (1.1 - 2.3)	0 - 10.4
end of follow-up	2.4 (1.6 - 3.3)	0 - 13.2

Table 2: Description of the LROI data set
