# Supplementary material for: CGR-CUSUM: a continuous time generalized rapid response cumulative sum chart
Source: Biostatistics. 2022 Sep 19;25(1):253–69. doi: 10.1093/biostatistics/kxac041 (PMC10939399; doi:10.1093/biostatistics/kxac041)
Supplement: kxac041_Supplementary_Data [file kxac041_supplementary_data.zip › kxac041-suppl_data/biosts-21444-File002.zip!/794524sctbvghvchmvmfcgjjvtqqqtwqrjkczn/Article/Lexis_CGR.pdf]

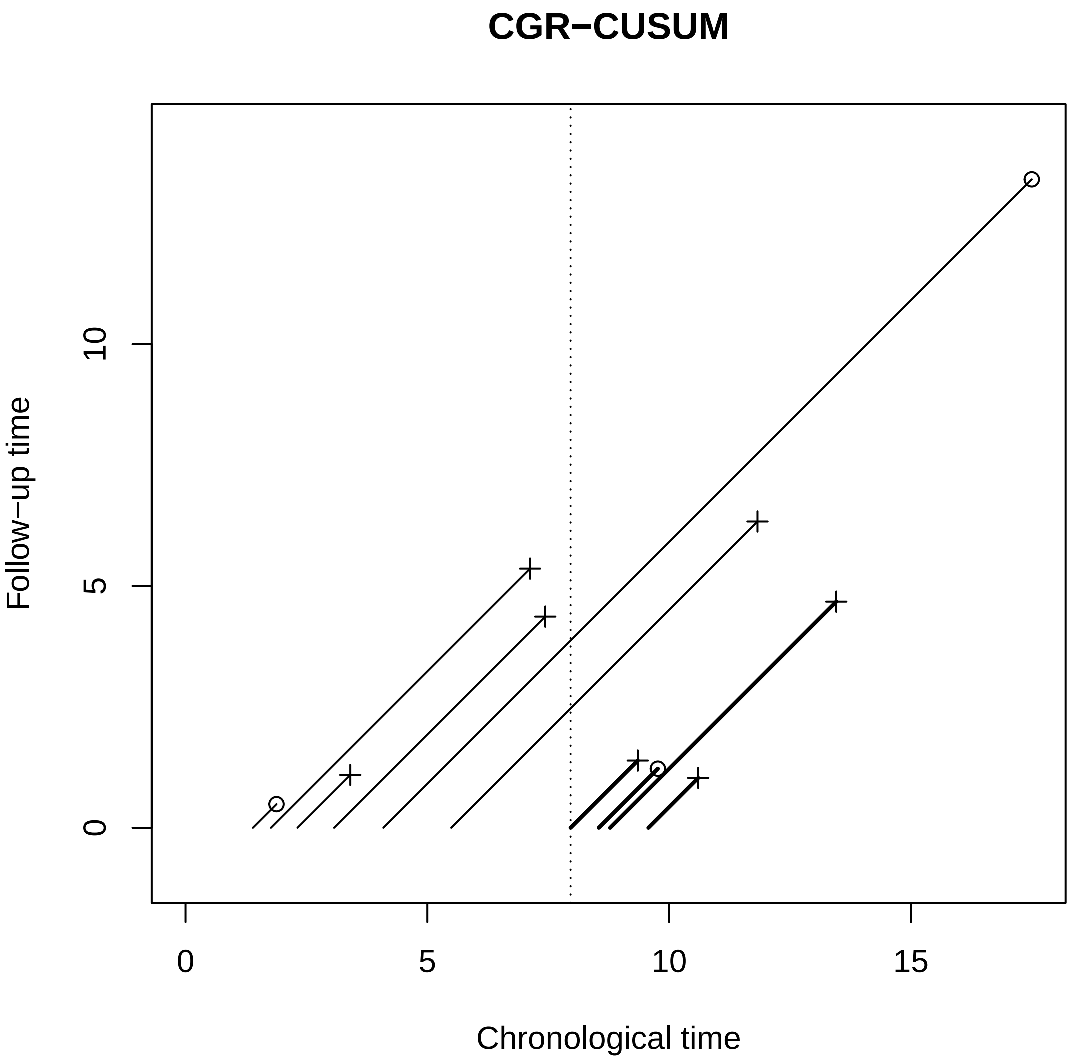
CGR−CUSUM
Follow−up time
Chronological time
0
5
10
15
0
5
10
